# Importance of cumulative exposure to elevated cholesterol and blood pressure in development of atherosclerotic coronary artery disease in systemic lupus erythematosus: a prospective proof-of-concept cohort study

**DOI:** 10.1186/ar3473

**Published:** 2011-09-29

**Authors:** Mandana Nikpour, Murray B Urowitz, Dominique Ibanez, Paula J Harvey, Dafna D Gladman

**Affiliations:** 1 University of Toronto Lupus Clinic and the Centre for Prognosis Studies in the Rheumatic Diseases, Toronto Western Hospital, 399 Bathurst Street, Toronto, ON, M5T 2S8, Canada; 2 The University of Melbourne Departments of Medicine and Rheumatology, St. Vincent's Hospital, 41 Victoria Parade, Fitzroy, Melbourne, Victoria, 3065, Australia; 3 Division of Cardiology, Women's College Hospital, 790 Bay Street, Suite 708, Toronto, ON, M5G 1N8 Canada

## Abstract

**Introduction:**

Previous studies have shown that traditional risk factors such as hypercholesterolemia and hypertension account for only a small proportion of the dramatically increased risk of atherosclerotic coronary artery disease (CAD) in systemic lupus erythematosus (SLE). However, in these studies, exposure to risk factors was measured only at baseline. In this study, our objective was to compare measures of cumulative exposure with remote and recent values for each of total cholesterol (TC), systolic (SBP), and diastolic (DBP) blood pressure in terms of ability to quantify risk of atherosclerotic CAD in patients with SLE.

**Methods:**

Patients in the Toronto lupus cohort had TC and BP measured at each clinic visit and were followed up prospectively for the occurrence of CAD. For each patient, arithmetic mean, time-adjusted mean (AM) and area-under-the-curve (AUC) were calculated for serial TC, SBP, and DBP measurements. Proportional hazards regression models were used to compare these summary measures with recent and first-available ("remote") measurements in terms of ability to quantify risk of CAD events, defined as myocardial infarction, angina, or sudden cardiac death.

**Results:**

The 991 patients had a mean ± SD of 19 ± 19 TC measurements per patient. Over a follow-up of 6.7 ± 6.4 years, 86 CAD events occurred; although remote TC was not significantly predictive of CAD, mean and AM TC were more strongly predictive (hazard ratio (HR) 2.07; *P *= 0.003) than recent TC (HR 1.86, *P *= 0.001). AUC TC was not predictive of CAD. A similar pattern was seen for DBP and SBP. Older age, male sex, higher baseline and recent disease activity score, and corticosteroid use also increased CAD risk, whereas antimalarials were protective.

**Conclusions:**

In contrast to the population-based Framingham model, first-available TC and BP are not predictive of CAD among patients with SLE, in whom measures reflecting cumulative exposure over time are better able to quantify CAD risk. This is an important consideration in future studies of dynamic risk factors for CAD in a chronic relapsing-remitting disease such as SLE. Our findings also underpin the importance of adequate control of SLE disease activity while minimizing corticosteroid use, and highlight the cardioprotective effect of antimalarials.

## Introduction

Systemic lupus erythematosus (SLE) is associated with a dramatically increased risk of atherosclerotic coronary artery disease (CAD), such that women with SLE aged 34 to 44 years are more than 50 times more likely to develop myocardial infarction (MI) than are age-matched peers [1]. Traditional risk factors measured at baseline, as defined in the Framingham model, do not fully account for this increased risk [2].

In the general population, it has been shown that recent and remote blood pressure (BP) predict cardiovascular risk incrementally over current BP [3]. In an inception cohort of patients with SLE, those with sustained hypercholesterolemia in the first 3 years of their disease were shown to be at greatest risk of cardiovascular events over 12 to 14 years of follow-up, compared with those who had persistently normal cholesterol or "variable" hypercholesterolemia in the first 3 years of disease [4]. We previously showed that both TC and BP take a variable course in patients with SLE and that almost half of the total variance over time in both TC and BP is seen within rather than between patients [5]. These findings suggest that the risk of future coronary events might be best quantified by using strategies that take into account multiple measurements of risk factors over time.

In this prospective proof-of-concept cohort study, we sought to compare "summary measures" of cumulative exposure to TC, SBP, and DBP, with single-point-in-time measurements of these risk factors (both recent and remote), in terms of ability to quantify CAD risk.

## Materials and methods

### Patients

Patients attending the University of Toronto lupus clinic are routinely seen at 2- to 6-monthly intervals wherein clinical and laboratory data, including TC, systolic blood pressure (SBP), and diastolic blood pressure (DBP) levels are obtained and recorded according to a set protocol. Patients are followed up prospectively for the occurrence of CAD events. In this study, we included patients who had two or more measurements of TC, SBP, and DBP taken before a CAD event (or last visit), in whom the gap between measurements did not exceed 18 months. Patients with a history of CAD before the start of the study were excluded. All patients fulfilled four or more of the American College of Rheumatology classification criteria for SLE, or had three criteria and a typical lesion of SLE on skin or renal biopsy [6,7]. Informed consent was obtained from all participants, and the study was approved by the research ethics board of the University Health Network.

### Methods

#### Measurement of TC, SBP, and DBP

Each measurement of TC, SBP, and DBP was tied to a clinic visit. TC was measured in plasma by using a commercial assay (Boehringer Mannheim kit 236691, Indianapolis, IN, USA) and recorded in millimoles per liter. As only small, clinically insignificant differences in TC are found when measured in the fasting or nonfasting state, nonfasting samples were used [8]. SBPs and DBPs were measured in millimeters of mercury (mm Hg) at every visit by using a manual sphygmomanometer. The patient was allowed to rest for 5 minutes in the sitting position. The reading was taken on the right arm, supported at the level of the heart. Korotkoff phase V (disappearance) was recorded as DBP.

#### Calculation of "summary measures" of TC, SBP, and DBP

For each of TC, SBP, and DBP, an arithmetic mean of all available measurements in each patient was calculated as the sum of all individual measurements divided by the total number of measurements. In each patient, for each of TC, SBP, and DBP, a time-adjusted mean (AM) was also calculated by using the formula:

$$\frac{\overset{n}{\underset{i=2}{\sum }}\left(\frac{x_{i}+x_{i-1}}{2}\right)t_{i}}{\overset{n}{\underset{i=2}{\sum }}t_{i}}$$

where x_i _is the level of the variable at visit I, and t_i _is the time interval between visit i and i-1. By incorporating the time interval between measurements in its calculation, the AM takes into account the length of time that TC, SBP, and DBP are presumed to have remained at a particular level. The arithmetic mean and AM were reported in millimoles per liter for TC and millimeters of mercury for SBP and DBP. In each patient, for each of TC, SBP, and DBP, the area-under-the-curve (AUC) was calculated by using integral calculus. AUC is reported in millimoles per liter multiplied by *t *and millimeters mercury multiplied by *t *for each of TC and BP, respectively, where *t *is the unit of time, in this case, months. For any given visit (V_i_), the summary measure for each of TC, SBP, and DBP was calculated from the first study visit (V_1_) up to and including the visit before (V_i-1_), thus ensuring that for all time intervals, exposure preceded outcome. The last visit (V_L_) was either a visit at which a CAD event was recorded or the last clinic visit as of August 2008 in those who remained CAD free.

#### Covariates

Covariates included in the proportional hazards models were sex, age, disease duration, disease-activity score (Systemic Lupus Erythematosus Disease Activity Index 2000; SLEDAI-2K), anti-phospholipid antibodies, "other" classic cardiovascular risk factors (diabetes and smoking), medications including corticosteroids, antimalarials, immunosuppressives, antihypertensives, and lipid-lowering therapy (statins), all recorded at baseline, and at each and every visit. Age and disease duration (from diagnosis to V_i_) were reported in years. SLEDAI-2K is an organ-weighted index of disease activity, scored from 0 to 105, with higher scores indicating more-active disease [9]. Antimalarials included chloroquine and hydroxychloroquine. Immunosuppressives included methotrexate, azathioprine, mycophenolate mofetil, cyclosporine, and cyclophosphamide. Antihypertensives included diuretics, β-blockers, calcium channel blockers, angiotensin-converting enzyme inhibitors, and angiotensin type II receptor blockers. The cumulative corticosteroid dose over the period of follow-up from study entry (V_1_) to the last visit (V_L_) was calculated and reported in grams. Use of all other medications was reported categorically at each visit, irrespective of dose. Diabetes was defined as fasting plasma glucose > 7.0 mmol/L or diabetes therapy ever. Current smoking was defined as smoking an average of one or more cigarette/s per day in the past month.

#### Outcome variables

CAD events were angina pectoris, myocardial infarction (MI), and sudden cardiac death. MI was defined as one of definite electrocardiographic (ECG) abnormalities, or typical symptoms with probable ECG abnormalities and abnormal enzymes (≥2 times upper limit of normal), or typical symptoms and abnormal enzymes. Angina pectoris was defined as severe pain or discomfort over the upper or lower sternum or anterior left chest and left arm, of short duration, relieved by rest or vasodilators in the absence of active SLE, or in the presence of atherosclerotic vascular disease elsewhere (for example, atherosclerotic peripheral vascular or cerebrovascular disease). The diagnosis of angina and MI required confirmation by a cardiologist. Sudden cardiac death was defined as death with undetermined cause but presumed cardiac.

A CAD event that occurred between V_i _and V_i+1 _was recorded at V_i+1_. For patients who had more than one CAD event, only the first was used in analysis. Some patients may have had both angina and MI recorded for the first time at a particular visit; this was treated as only one event rather than two.

#### Univariate comparisons

For each of the TC and BP models, univariate comparisons of demographic, disease- and treatment-related variables and traditional cardiac risk factors in patients who had CAD events and those that remained CAD free were performed by using *t *tests for continuous variables and χ^2 ^tests for categoric variables. In case of non-normally distributed data, Mann-Whitney *U *tests were used for continuous variables. Two-sided *P *values (*P*) ≤ 0.05 were considered to be significant.

#### Time-constant regression models

For each of TC, SBP, and DBP, two time-constant proportional hazards models were run. Variables in the first model included first available (baseline) measurement of TC (or SBP, DBP), along with sex, age, SLEDAI-2K score at study entry (V_1 _or "baseline"), and anti-phospholipid antibodies, diabetes, smoking, corticosteroid, antimalarial, immunosuppressive, antihypertensive, and lipid-lowering medication use "ever" from V_1 _to V_L-1_.

The second model included the average (arithmetic mean) of the first two available measurements of TC (or SBP or DBP) and all covariates included in the first Cox model.

#### Time-dependent regression models

For each of TC, SBP, and DBP, we also ran four time-dependent proportional hazards regression models. In the first model, recent measurements of TC (or SBP or DBP) were used in a dynamic manner, varying from visit to visit. In the remaining three models, summary measures (mean, AM, AUC) were used in a time-dependent manner (that is, updated from visit to visit). Covariates in these models included sex, age, SLEDAI-2K score, anti-phospholipid antibodies, diabetes, smoking, corticosteroid, antimalarial, immunosuppressive, anti-hypertensive and lipid-lowering medication use, also treated in a time-dependent fashion (that is, updated from visit to visit). For each of TC, SBP, and DBP at each visit, single point, mean, and AM measurements were strongly correlated. Therefore, each summary measure was analyzed in a separate model.

Both time-constant and time-dependent models are reported as hazard ratios (HRs) with accompanying 95% confidence interval (95% CI) and *P *value, for each of the predictor variables and covariates. All statistical analyses were performed by using the SAS software version 9.1 (SAS Institute, Inc., Cary, NC, U.S.A.).

## Results

Characteristics of the patients in this study are presented in Table 1. Overall, the BP dataset contained 991 patients, whereas the TC dataset comprised 956 patients. In each dataset, patients were mostly female (88%) and mostly Caucasian (70%). A total of 94 coronary events occurred (75 angina, 25 MI, and two sudden cardiac deaths; eight had both angina and MI) in the BP dataset, whereas the TC dataset contained 86 coronary events (71 angina, 20 MI, and two sudden cardiac deaths; seven had both angina and MI). The mean ± SD age and disease duration at entry into the study were very similar for both datasets (37.1 ± 14.0 and 6.1 ± 7.9 years, respectively, for the BP dataset). Likewise, mean ± SD SLEDAI-2K score and Systemic Lupus Erythematosus International Collaborating Clinics/American College of Rheumatology Damage Index (SLICC/ACR-DI) at study entry were similar in the two datasets (9.2 ± 7.5 and 0.5 ± 1.2, respectively, for the BP dataset), indicating moderate disease activity and minimal disease-related damage [10]. For each dataset, at entry into the study, more than 60% of patients were taking corticosteroids, whereas approximately 40% were taking antimalarials, and 25% were taking immunosuppressives. In each dataset, at the start of the study, approximately 22% of patients were hypertensive, 40% had hypercholesterolemia, 3% had diabetes, and 19% were smokers. At study start, in each dataset, 25% were taking antihypertensives, and 5% were taking lipid-lowering medications.

**Table 1:** Patient characteristics

	BP Dataset	TC Dataset
	*n *(%) or mean ± SD	*n *(%) or mean ± SD
Number of patients	991	956
Female	879 (88.7%)	849 (88.8%)
Race		
Caucasian	70%	70%
Black	11%	11%
Asian	11%	11%
Other	8%	8%
CAD events		
MI Angina	25 75	20 71
Sudden cardiac death	2	2
Total	94	86
Age^a^		
At diagnosis	31.0 ± 13.8	30.9 ± 13.8
At study entry	37.1 ± 14.0	37.5 ± 14.1
Disease duration^a^		
At first clinic visit	4.0 ± 5.8	4.0 ± 5.9
At study entry	6.1 ± 7.9	6.6 ± 8.1
SLEDAI-2K^b^		
At first clinic visit	9.7 ± 7.7	9.6 ± 7.7
At study entry	9.2 ± 7.5	8.3 ± 7.2
SLICC/ACR-DI^c^		
At first clinic visit	0.3 ± 0.7	0.3 ± 0.7
At study entry	0.5 ± 1.2	0.6 ± 1.2
Steroids at study entry	623 (63%)	625 (65.6%)
Antimalarials at study entry^d^	380 (38.5%)	385 (40.4%)
Immunosuppressives^e^		
At study entry	235 (23.8%)	233 (24.5%)
Hypertension at study entry^f^	221 (22.3%)	212 (22.6%)
Hypercholesterolemia at study entry^g^	344 (41.8%)	408 (42.7%)
Diabetes at study entry^h^	28 (2.9%)	31 (3.3%)
Smoker at study entry^i^	190 (19.5%)	183 (19.5%)
Antihypertensive use		
at study entry^j^	174/691 (25.2%)	174/699 (24.9%)
Lipid-lowering meds		
at study entry^k^	23/440 (5.2%)	22/440 (5.0%)

BP, blood pressure (mm Hg); CAD, coronary artery disease; MI, myocardial infarction; SD, standard deviation; SLEDAI-2K, Systemic Lupus Erythematosus Disease Activity Index 2000; SLICC/ACR-DI, Systemic Lupus International Collaborating Clinics/American College of Rheumatology Damage Index; TC, total plasma cholesterol (mmol/L). ^a^Years. ^b^Scores range from 0 to 105, with higher scores indicating more-active disease. ^c^Scores range from 0 to 46, with higher scores indicating greater disease-related damage. ^d^Antimalarials include chloroquine and hydroxychloroquine. ^e^Immunosuppressives include methotrexate, azathioprine, mycophenolate mofetil, cyclosporine, and cyclophosphamide. ^f^Diastolic BP ≥90 or systolic BP ≥140 mm Hg or treatment with antihypertensive medication. ^g^Hypercholesterolemia was defined as cholesterol > 5.2 mmol/L or lipid-lowering therapy. ^h^Diabetes was defined as fasting plasma glucose > 7.0 mmol/L or diabetes therapy. ^i^Current smoking is defined as smoking an average of one or more cigarettes per day over the past month. ^j^All classes of antihypertensives including diuretics, β-blockers, calcium channel blockers, angiotensin-converting enzyme inhibitors, and angiotensin type II receptor blockers. ^k^HMG Co-A reductase inhibitors (statins)

### Summary measures for BP

The calculation of summary measures was based on 19, 579 individual measurements of SBP and DBP, with a mean ± SD of 20 ± 20 (median, 13) serial measurements per patient. The mean ± SD (median) time interval between measurements was 4.2 ± 2.3 (3.4) months. The mean ± SD (median) time from study start to the visit before a CAD event (or last clinic visit) was 6.5 ± 6.7 (4.2) years. The mean ± SD (median) length of follow-up from study start to CAD event (or last clinic visit) was 7.0 ± 6.7 (4.6) years. Among all patients, the mean SBP at the start of study was 123.9 ± 19.4 mm Hg, whereas the mean DBP at the start of study was 77.6 ± 12.3 mm Hg.

### Summary measures for TC

The calculation of summary measures was based on 17, 936 individual measurements of TC, with a mean ± SD of 19 ± 19 (median, 12) serial measurements per patient. The mean ± SD (median) time interval between TC measurements was 4.3 ± 2.3 (3.6) months. The mean ± SD (median) time from study start to the visit before a CAD event (or last clinic visit) was 6.3 ± 6.4 (4.2) years. The mean ± SD (median) length of follow-up from study start to CAD event (or last clinic visit) was 6.7 ± 6.4 (4.6) years. Among all patients, the mean TC level at the start of study was 5.3 ± 1.6 mmol/L.

### Univariate comparisons

Univariate comparisons of patients with and without a CAD event for each of the BP and TC models are presented in Table 2. In the BP models, patients who experienced CAD events were more likely to be Caucasian (87.2% versus 68.6%; *P *= 0.004), older (46.0 ± 13.2 versus 36.6 ± 13.9 years; *P *< 0.0001), menopausal (44.4% versus 23.7%; *P *= 0.0001), hypertensive (31.0% versus 14.4%; *P *< 0.0001), and taking corticosteroids (76.7% versus 68.5%; *P *= 0.02) at the start of the study. Patients with CAD events were also more likely to have been older at lupus diagnosis (39.0 ± 14.1 versus 30.1 ± 13.5 years; *P *< 0.0001) and to have anti-phospholipid antibodies (75.8% versus 61.1%; *P *= 0.006), hypertension (82.6% versus 43.7%; *P *< 0.0001), hypercholesterolemia (91.9% versus 68.6%; *P *< 0.0001), and diabetes mellitus (15.1% versus 6.7%; *P *= 0.005) during follow-up, than were those who remained CAD free. In addition, they were more likely to be treated with corticosteroids (94.2% versus 77.7%; *P *= 0.0003) at higher cumulative doses (42.3 ± 34.4 versus 31.7 ± 34.7 g; *P *= 0.006), antihypertensives (90.8% versus 72.9%; *P *= 0.0008), and lipid-lowering medications (61.8% versus 26.5%; *P *< 0.0001) during follow-up. Patients with CAD events were less likely to have been exposed to antimalarials during follow-up (59.3% versus 71.5%; *P *= 0.02).

**Table 2:** Univariate comparison of patients with and without CAD used in TC and BP models

	BP models			TC models		
	CAD (*n *= 94)	No CAD (*n *= 897)		CAD (*n *= 86)	No CAD (*n *= 870)	
	*n*(%) or mean ± SD	*n*(%) or mean ± SD	*P*	*n*(%) or mean ± SD	*n*(%) or mean ± SD	*P*
Female	72 (83.7%)	777 (89.3%)	0.12	78 (83.0%)	801 (89.3%)	0.07
Menopause^a ^at study start	32 (44.4%)	184 (23.7%)	0.0001	33 (42.3%)	189 (23.6%)	0.0003
Race						
Caucasian	75 (87.2%)	574 (68.6%)	0.004	81 (86.2%)	593 (68.7%)	0.005
Black	3 (3.5%)	96 (11.5%)		4 (4.3%)	97 (11.2%)	
Asian	4 (4.7%)	94 (11.2.%)		4 (4.3%)	97 (11.2%)	
Other	4 (4.7%)	73 (8.7%)		5 (5.3%)	76 (8.8%)	
Disease duration^b^						
At first visit	4.08 ± 5.71	4.01 ± 5.87	0.92	4.24 ± 5.72	3.94 ± 5.80	0.63
At study start	6.99 ± 7.84	6.54 ± 8.17	0.62	5.95 ± 7.23	6.13 ± 8.00	0.83
SLEDAI-2K^c^						
At first visit	12.34 ± 8.67	9.49 ± 7.63	0.001	12.16 ± 8.63	9.60 ± 7.65	0.002
At study start	9.59 ± 7.99	8.28 ± 7.26	0.11	11.36 ± 8.86	9.02 ± 7.50	0.02
Disease manifestations^d^ ever from diagnosis to first visit						
Musculoskeletal	42 (44.7%)	365 (40.7%)	0.45	44 (51.2%)	363 (41.7%)	0.09
Cutaneous	69 (73.4%)	504 (56.2%)	0.001	65 (75.6%)	507 (58.3%)	0.002
Renal	46 (48.9%)	364 (40.6%)	0.12	47 (54.7%)	370 (42.5%)	0.03
Nervous system	32 (34.0%)	180 (20.1%)	0.002	32 (37.2%)	189 (21.7%)	0.001
Hematologic	13 (13.8%)	110 (12.3%)	0.66	14 (16.3%)	117 (13.5%)	0.47
Vasculitis	19 (20.2%)	126 (14.1%)	0.11	19 (22.1%)	133 (15.3%)	0.10
Immunologic	58 (61.7%)	640 (71.4%)	0.05	62 (72.1%)	637 (73.2%)	0.82
Serosal	13 (13.8%)	77 (8.6%)	0.09	11 (12.8%)	74 (8.5%)	0.18
Fever	16 (17.0%)	141 (15.7%)	0.74	18 (20.9%)	138 (15.9%)	0.23
Disease manifestations^e ^ever during follow-up						
Musculoskeletal	70 (74.5%)	508 (56.6%)	0.0008	67 (77.9%)	476 (54.7%)	< 0.0001
Cutaneous	82 (87.2%)	643 (71.7%)	0.001	73 (84.9%)	615 (70.7%)	0.005
Renal	72 (76.6%)	581 (64.8%)	0.02	62 (72.1%)	547 (62.9%)	0.09
Nervous system	58 (61.7%)	304 (33.9%)	< 0.0001	53 (61.6%)	284 (32.6%)	< 0.0001
Hematologic	25 (26.6%)	229 (25.5%)	0.82	22 (25.6%)	214 (24.6%)	0.84
Vasculitis	42 (44.7%)	207 (23.1%)	< 0.0001	36 (41.9%)	189 (21.7%)	< 0.0001
Immunologic	82 (87.2%)	769 (85.7%)	0.69	76 (88.4%)	741 (85.2%)	0.42
Serosal	22 (23.4%)	112 (12.5%)	0.003	19 (22.1%)	94 (10.8%)	0.002
Fever	42 (44.7%)	231 (25.8%)	< 0.0001	40 (46.5%)	200 (23.0%)	< 0.0001
Chronic renal insufficiency ever^f^	5 (5.3%)	64 (7.1%)	0.51	4 (4.7%)	60 (6.9%)	0.43
Anti-phospholipid antibodies^g^						
Ever from first clinic visit to last visit	69 (75.8%)	546 (61.1%)	0.006	66 (76.7%)	527 (60.8%)	0.004
Ever in the study period	66 (72.5%)	491 (55.0%)	0.001	61 (70.9%)	459 (53.0%)	0.001
Corticosteroids						
At study start	66 (76.7%)	559 (68.5%)	0.02	66 (70.2%)	557 (62.2%)	0.13
Ever during follow-up	81 (94.2%)	676 (77.7%)	0.0003	88 (93.6%)	706 (78.7%)	0.0006
Cumulative steroid dose (g)						
From first clinic visit to last visit	42.3 ± 34.4	31.7 ± 34.7	0.006	43.5 ± 34.6	31.9 ± 35.1	0.005
In the study period	30.3 ± 25.8	20.5 ± 23.1	0.0002	28.8 ± 25.1	19.7 ± 21.9	0.0005
Antimalarials^g^						
At study start	29 (33.7%)	356 (41.1%)	0.18	31 (33.0%)	349 (39.0%)	0.25
Ever during follow-up	51 (59.3%)	622 (71.5%)	0.02	58 (61.7%)	639 (71.2%)	0.05
Immunosuppressives^h^						
At study start	16 (18.6%)	217 (25.1%)	0.18	14 (15.1%)	221 (24.8%)	0.04
Ever during follow-up	46 (53.5%)	454 (52.2%)	0.82	51 (54.3%)	471 (52.5%)	0.75
Hypertension^i^						
At study start	26 (31.0%)	123 (14.4%)	< 0.0001	26 (27.7%)	131 (14.6%)	0.001
Ever during follow-up	71 (82.6%)	380 (43.7%)	< 0.0001	77 (81.9%)	403 (44.9%)	< 0.0001
Hypercholesterolemia^j^						
At study start	26 (31.0%)	350 (40.2%)	< 0.0001	39 (65.0%)	305 (39.9%)	0.0001
Ever during follow-up	79 (91.9%)	597 (68.6%)	< 0.0001	81 (91.0%)	614 (69.1%)	< 0.0001
Diabetes mellitus^k^						
At study start	5 (6.0%)	29 (3.4%)	0.22	4 (4.4%)	27 (3.1%)	0.52
Ever during follow-up	13 (15.1%)	58 (6.7%)	0.005	13 (13.8%)	61 (6.8%)	0.01
Smoker^l^						
At study start	20 (23.8%)	162 (19.0%)	0.28	23 (25.0%)	166 (18.8%)	0.15
Ever during follow-up	27 (31.4%)	220 (25.4%)	0.22	30 (31.9%)	229 (25.6%)	0.18
Antihypertensives^m^						
Ever up to study start	14/37 (37.8%)	166/642 (25.9%)	0.11	16/39 (41.0%)	169/654 (25.8%)	0.04
Ever during follow-up	69/76 (90.8%)	355/487 (72.9%)	0.0008	71/83 (85.5%)	361/506 (71.3%)	0.007
Lipid-lowering medications^n^						
Ever up to study start	1/10 (10.0%)	26/427 (6.1%)	0.48	1/11 (9.1%)	27/430 (6.3%)	0.52
Ever during follow-up	47/76 (61.8%)	144/543 (26.5%)	< 0.0001	48/70 (68.6%)	145/528 (27.5%)	< 0.0001
TC level at study start^o^	5.85 ± 1.62	5.19 ± 1.56	0.0002			
Mean of first two TC levels^o^	5.91 ± 1.53	5.19 ± 1.49	< 0.0001			
Mean of all TC levels^o^	5.72 ± 1.23	4.95 ± 1.11	< 0.0001			
AM of all TC levels^o^	5.72 ± 1.23	4.94 ± 1.11	< 0.0001			
AUC of all TC levels^p^	15, 806 ± 13, 063	11, 117 ± 11, 976	0.0006			
SBP at study start^o^				131.65 ± 21.29	123.11 ± 19.08	< 0.0001
Mean of first two SBPs^o^				132.45 ± 19.00	123.03 ± 17.35	< 0.0001
Mean of all SBPs^o^				134.90 ± 15.32	121.96 ± 14.81	< 0.0001
AM of all SBPs^o^				134.68 ± 15.351	121.88 ± 14.99	< 0.0001
AUC of all SBPs^∞p^				400, 217 ± 318, 056	285, 335 ± 304, 573	0.0006
DBP at study start^o^				77.3 ± 12.3	80.8 ± 11.8	0.009
Mean of first two DBPs^o^				77.2 ± 10.5	82.0 ± 10.5	< 0.0001
Mean of all DBPs^o^				76.2 ± 8.5	82.7 ± 6.7	< 0.0001
AM of all DBPs^o^				76.2 ± 8.6	82.6 ± 6.8	< 0.0001
AUC of all DBPs^p^				179, 476 ± 192, 071	245, 236 ± 194, 324	0.002

AM, time-adjusted mean; AUC, area-under-the-curve; CAD, coronary artery disease; DBP, diastolic blood pressure; SBP, systolic blood pressure; SLEDAI-2K, Systemic Lupus Erythematosus Disease Activity Index 2000; TC, total plasma cholesterol (mmol/L). ^a^Menopause defined as a minimum of 12 months of amenorrhea irrespective of cause. ^b^Years. ^c^Scores range from 0 to 105, with higher scores indicating more-active disease. ^d^Disease manifestations defined based on SLEDAI-2K definitions. ^e^Chronic renal insufficiency defined based on SLICC/ACR DI definition (glomerular filtration rate < 50%, proteinuria ≥3.5 g/24 h, or end-stage renal disease). ^f^Measured by using commercial assays, with test reported positive if either IgM or IgG antibody level exceeded manufacturer-recommended cut points. ^g^Antimalarials include chloroquine and hydroxychloroquine. ^h^Immunosuppressives include methotrexate, azathioprine, mycophenolate mofetil, cyclosporine, and cyclophosphamide. ^i^Hypertension was defined as DBP ≥90 or SBP ≥140 mm Hg or treatment with antihypertensive medication. ^j^Hypercholesterolemia was defined as TC > 5.2 mmol/L or lipid-lowering therapy. ^k^Diabetes was defined as fasting plasma glucose > 7.0 mmol/L or diabetes therapy. ^l^Current smoking is defined as smoking of an average of one or more cigarettes per day over the past month. ^m^All classes of antihypertensives including diuretics, β-blockers, calcium channel blockers, angiotensin-converting enzyme inhibitors, and angiotensin type II receptor blockers. ^n^HMG Co-A reductase inhibitors (statins). ^o^mmol/L. ^p^mmol/L multiplied by months.

In the TC models, univariate comparison of patients with and without CAD outcomes revealed results that were similar to the BP models (Table 2). TC level at study start (5.85 ± 1.62 versus 5.19 ± 1.56 mmol/L; *P *= 0.0002), average of first two TC levels (5.91 ± 1.53 versus 5.19 ± 1.49 mmol/L; *P *< 0.0001), and mean (5.72 ± 1.23 versus 4.95 ± 1.11 mmol/L; *p *< 0.0001), AM (5.72 ± 1.23 versus 4.94 ± 1.11; *P *< 0.0001), and AUC (15, 806 ± 13, 063 versus 11, 1117 ± 11, 976 mmol/L; *P *= 0.0006) of all serial TC levels were higher in patients who had CAD events than in those who remained CAD free.

In both the BP and TC datasets, patients with CAD events were more likely to have musculoskeletal, cutaneous, renal, and nervous system manifestations of lupus and were also more likely to have vasculitis, serositis, and fever during the course of their disease. However, no difference was found in the prevalence of chronic renal insufficiency, based on SLICC/ACR DI definition [10], among those with and without CAD (5.3% versus 7.1%; *P *= 0.51 in the BP dataset).

### Proportional hazards multiple regression models

#### TC models

Table 3 shows the results of the proportional hazards models for CAD events by using various measures of TC. In the time-constant models (columns 1 and 2), neither first-available ("remote") TC nor the average of first two TC levels was significantly associated with a CAD event. However, in these models, male sex (HR = 2.02; *P *= 0.02), age (HR = 1.06; *P *< 0.0001), and SLEDAI-2K (HR = 1.03; *P *= 0.04) at study start (baseline), and steroid use ever (HR = 4.17; *P *= 0.003) were significantly associated with a CAD event. Antimalarial use ever was protective against CAD (HR = 0.50; *P *= 0.003).

**Table 3:** Proportional hazards models for coronary outcomes using various measures of cholesterol

	Time-constant models				Time-dependent models							
	First available ("remote") TC		Average of first two TCs		Most recent TC		Mean TC		Time-adjusted mean TC		AUC TC	
	HR (95% CI)	*P*	HR (95% CI)	*P*	HR (95% CI)	*P*	HR (95% CI)	*P*	HR (95% CI)	*P*	HR (95% CI)	*p*
Cholesterol (TC)	1.07 (0.93, 1.22)	0.36	1.08 (0.94, 1.24)	0.30	1.22 (1.05, 1.42)	0.01	1.22 (1.01, 1.47)	0.04	1.22 (1.02, 1.47)	0.03	1.00 (1.00, 1.00)	0.29
Male sex	2.02 (1.12, 3.62)	0.02	2.00 (1.11, 3.59)	0.02	1.84 (1.02, 3.32)	0.04	1.75 (0.97, 3.18)	0.06	1.74 (0.96, 3.15)	0.07	1.78 (0.98, 3.23)	0.06
Age^ψ^	1.06 (1.04, 1.08)	< 0.0001	1.06 (1.04, 1.08)	< 0.0001	1.06 (1.05, 1.08)	< 0.0001	1.06 (1.05, 1.08)	< 0.0001	1.06 (1.04, 1.08)	< 0.0001	1.06 (1.04, 1.08)	< 0.0001
SLEDAI-2K^¶^	1.03 (1.001, 1.06)	0.04	1.03 (1.00, 1.06)	0.06	1.09 (1.05, 1.13)	< 0.0001	1.09 (1.05, 1.13)	< 0.0001	1.09 (1.05, 1.13)	< 0.0001	1.09 (1.05, 1.13)	< 0.0001
Hypertension^¢^	1.12 (0.61, 2.04)	0.72	1.10 (0.60, 2.02)	0.76	1.50 (0.93, 2.42)	0.095	1.57 (0.97, 2.52)	0.06	1.57 (0.98, 2.52)	0.06	1.67 (1.05, 2.67)	0.03
Corticosteroids^β^	4.17 (1.62, 10.73)	0.003	4.16 (1.62, 10.72)	0.003	1.85 (1.05, 3.23)	0.03	1.89 (1.08, 3.30)	0.03	1.89 (1.08, 3.30)	0.03	1.92 (1.10, 3.35)	0.02
Antimalarials^§^	0.50 (0.31, 0.79)	0.003	0.50 (0.31, 0.80)	0.004	0.90 (0.57, 1.44)	0.66	0.88 (0.55, 1.41)	0.59	0.88 (0.55, 1.41)	0.60	0.80 (0.51, 1.27)	0.35
Immunosuppressives^¥^	0.79 (0.49, 1.26)	0.32	0.78 (0.49, 1.25)	0.30	1.32 (0.82, 2.14)	0.25	1.29 (0.79, 2.08)	0.31	1.29 (0.79, 2.09)	0.31	1.34 (0.83, 2.17)	0.23

95% CI, 95% confidence interval; AUC, area under the curve; BP, blood pressure (mm Hg); DBP, diastolic blood pressure (mm Hg); HR, hazard ratio; NS: not significant; *P, P *value; SBP, systolic blood pressure (mm Hg); TC, total plasma cholesterol (mmol/L).

^ψ^Years; age at the time of first available TC (or SBP or DBP) in time-constant models and coincident with each TC (or SBP or DBP) measurement in the time-dependent covariate models. ^¶^SLE Disease Activity Index 2000; scores range from 0 to 105, with higher scores indicating more-active disease; measured at the time of first available TC (or SBP or DBP) in time-constant models, and coincident with each TC (or SBP or DBP) measurement in the time-dependent covariate models. ^¢^Hypertension defined as diastolic BP ≥90 or systolic BP ≥140 mm Hg; defined as present ever during the study in time-constant models, and coincident with each TC measurement in the time-dependent covariate models. ^β^Corticosteroids defined as used ever during the study in time-constant models, and coincident with each TC (or SBP or DBP) measurement in the time-dependent covariate models. ^ζ^Elevated total plasma cholesterol (hypercholesterolemia) defined as total plasma cholesterol > 5.2 mmol/L; measured at the time of first available SBP or DBP in time-constant models, and coincident with each SBP or DBP measurement in the time-dependent covariate models. ^§^Antimalarials include chloroquine and hydroxychloroquine; defined as used ever during the study in time-constant models, and coincident with each TC (or SBP or DBP) measurement in the time-dependent covariate models. ^¥^Immunosuppressives include methotrexate, azathioprine, mycophenolate mofetil, cyclosporine, and cyclophosphamide; defined as used ever during the study in time-constant models, and coincident with each TC (or SBP or DBP) measurement in the time-dependent covariate models.

In time-dependent models (Table 3, columns 3 to 5, inclusive), most *recent *(HR = 1.22; *P *= 0.01), *mean *(HR = 1.22; p = 0.04) and *AM *(HR = 1.22; p = 0.03) TC at each visit were significantly associated with CAD event. When only the significant covariates were included in the models (Table 4), *mean *and *AM *TC were more strongly predictive of CAD (HR = 2.07; *P *= 0.003 for both) than was recent TC (HR = 1.86; *P *= 0.001). In these models, other significant covariates included male sex (HR = 1.84; *P *= 0.04, in *recent *TC model), age (HR = 1.06; *P *< 0.0001 for all three models), SLEDAI-2K score (HR = 1.09; *P *< 0.0001 for all three models), steroid use (HR = 1.85 and 1.89 for *mean *and *AM *TC, *P *= 0.03 for all three models) and hypertension at each visit (HR = 1.57; *P *= 0.06 for both *mean *and *AM *TC models). *AUC *TC (Table 3, last column) was not significantly associated with CAD.

**Table 4:** Proportional hazards models for coronary outcomes using various measures of cholesterol, including only significant covariates in the models

	Time-dependent covariate models					
	**Most recent TC**		**Mean TC**		**Time-adjusted** **mean TC**	
	HR (95% CI)	*P*	HR (95% CI)	*P*	HR (95% CI)	*P*
Cholesterol (TC)	1.86 (1.30, 2.68)	0.001	2.07 (1.28, 3.36)	0.003	2.07 (1.28, 3.34)	0.003
Male sex	1.90 (1.06, 3.41)	0.03	1.83 (1.01, 3.29)	0.046	1.82 (1.01, 3.29)	0.047
Age^ψ^	1.12 (1.07, 1.17)	< 0.0001	1.13 (1.06, 1.20)	< 0.0001	1.13 (1.06, 1.20)	< 0.0001
SLEDAI-2K^¶^	1.09 (1.05, 1.13)	< 0.0001	1.10 (1.06, 1.14)	< 0.0001	1.10 (1.06, 1.14)	< 0.0001
Corticosteroids^β^	2.04 (1.20, 3.45)	0.01	2.01 (1.19, 3.41)	0.01	2.01 (1.19, 3.41)	0.01

95% CI, 95% confidence interval; AUC, area under the curve; BP, blood pressure (mm Hg); DBP: diastolic blood pressure (mm Hg); HR, hazard ratio; NS, not significant; *P, P *value; SBP, systolic blood pressure (mm Hg); TC, total plasma cholesterol (mmol/L).

^ψ^Years; age at the time of first available TC (or SBP or DBP) in time-constant models and coincident with each TC (or SBP or DBP) measurement in the time-dependent covariate models. ^¶^SLE Disease Activity Index 2000; scores range from 0 to 105, with higher scores indicating more-active disease; measured at the time of first available TC (or SBP or DBP) in time-constant models, and coincident with each TC (or SBP or DBP) measurement in the time-dependent covariate models. ^¢^Hypertension defined as diastolic BP ≥ 90 or systolic BP ≥ 140 mm Hg; defined as present ever during the study in time-constant models, and coincident with each TC measurement in the time-dependent covariate models. ^β^Corticosteroids defined as used ever during the study in time-constant models, and coincident with each TC (or SBP or DBP) measurement in the time-dependent covariate models. ^ζ^Elevated total plasma cholesterol (hypercholesterolemia) defined as total plasma cholesterol > 5.2 mmol/L; measured at the time of first available SBP or DBP in time-constant models, and coincident with each SBP or DBP measurement in the time-dependent covariate models. ^§^Antimalarials include chloroquine and hydroxychloroquine; defined as used ever during the study in time-constant models, and coincident with each TC (or SBP or DBP) measurement in the time-dependent covariate models. ^¥^Immunosuppressives include methotrexate, azathioprine, mycophenolate mofetil, cyclosporine, and cyclophosphamide; defined as used ever during the study in time-constant models, and coincident with each TC (or SBP or DBP) measurement in the time-dependent covariate models.

### SBP models

Results of the proportional hazards models for CAD outcomes by using various measures of systolic blood pressure (SBP) are presented in Table 5. In the time-constant models (columns 1 and 2), neither first available (remote) SBP nor the average of first two SBPs was associated with a CAD event. However, in these models, male sex (HR = 2.01; *P *= 0.02 for first-available SBP model; HR = 2.04; *P *= 0.02 for average-of-first-two SBP model), age (HR = 1.05; *P *< 0.0001) and SLEDAI-2K (HR = 1.03; *P *= 0.01) at baseline, and steroid use ever (HR = 2.73 for first available and 2.74 for average of first two SBPs, *P *= 0.03) were significantly associated with a CAD event. Antimalarial use ever was protective against CAD (HR = 0.59; *P *= 0.02). Disease duration, elevated TC, and immunosuppressive use at baseline were not significantly associated with CAD.

**Table 5:** Proportional hazards models for coronary events using various measures of systolic blood pressure

	Time-constant models				Time-dependent models							
	First available ("remote") SBP		Average of first two SBP		Most recent SBP		Mean SBP		Time-adjusted mean SBP		AUC SBP	
	HR (95% CI)	*P*	HR (95% CI)	*P*	HR (95% CI)	*P*	HR (95% CI)	*P*	HR (95% CI)	*P*	HR (95%)I)	*p*
Systolic blood pressure (SBP)	1.00 (0.99, 1.02)	0.45	1.31 (0.68, 2.53)	0.42	1.01 (1.00, 1.02)	0.052	1.025 (1.01, 1.04)	0.004	1.024 (1.01, 1.04)	0.004	1.00 (1.00, 1.00)	0.19
Male sex	2.01 (1.13, 3.59)	0.02	2.04 (1.14, 3.65)	0.02	1.87 (1.03, 3.39)	0.04	1.75 (0.96, 3.21)	0.07	1.74 (0.95, 3.18)	0.07	1.83 (1.00, 3.33)	0.049
Age^ψ^	1.05 (1.03, 1.07)	< 0.0001	1.05 (1.03, 1.07)	< 0.0001	1.06 (1.03, 1.07)	< 0.0001	1.04 (1.02, 1.07)	< 0.0001	1.04 (1.02, 1.07)	< 0.0001	1.06 (1.04, 1.08)	< 0.0001
SLEDAI-2K^¶^	1.03 (1.01, 1.06)	0.01	1.03 (1.01, 1.06)	0.01	1.09 (1.05, 1.13)	< 0.0001	1.09 (1.05, 1.13)	< 0.0001	1.09 (1.05, 1.13)	< 0.0001	1.09 (1.05, 1.13)	< 0.0001
Elevated cholesterol^ζ^	1.41 (0.67, 2.98)	0.37	1.39 (0.66, 2.95)	0.39	1.72 (1.06, 2.78)	0.03	1.68 (1.04, 2.71)	0.03	1.69 (1.04, 2.73)	0.03	1.82 (1.13, 2.92)	0.01
Corticosteroids^β^	2.73 (1.13, 6.61)	0.03	2.74 (1.13, 6.62)	0.03	1.74 (0.99, 3.08)	0.06	1.72 (0.98, 3.03)	0.06	1.72 (0.97, 3.03)	0.06	1.77 (1.00, 3.12)	0.049
Antimalarials^§^	0.59 (0.37, 0.93)	0.02	0.59 (0.37, 0.95)	0.03	0.95 (0.60, 1.52)	0.84	0.97 (0.61, 1.56)	0.91	0.97 (0.61, 1.55)	0.89	0.93 (0.58, 1.48)	0.75
Immunosuppressives^¥^	0.91 (0.58, 1.43)	0.68	0.89 (0.56, 1.41)	0.63	1.51 (0.93, 2.45)	0.10	1.48 (0.91, 2.40)	0.11	1.48 (0.91, 2.40)	0.11	1.46 (0.90, 2.36)	0.13

95% CI: 95% confidence interval; AUC: area under the curve; BP: blood pressure (mmHg); DBP: diastolic blood pressure (mmHg); HR: hazard ratio; NS: not significant; *p: *p value; SBP: systolic blood pressure (mmHg); TC: total plasma cholesterol (mmol/L).

^ψ^Years; age at the time of first available TC (or SBP or DBP) in time-constant models and coincident with each TC (or SBP or DBP) measurement in the time-dependent covariate models. ^¶^SLE Disease Activity Index 2000; scores range from 0 to 105, with higher scores indicating more active disease; measured at the time of first available TC (or SBP or DBP) in time-constant models, and coincident with each TC (or SBP or DBP) measurement in the time-dependent covariate models. ^¢^Hypertension defined as Diastolic BP ≥ 90 or Systolic BP ≥ 140 mmHg; defined as present ever during the study in time-constant models, and coincident with each TC measurement in the time-dependent covariate models. ^β^Corticosteroids defined as used ever during the study in time-constant models, and coincident with each TC (or SBP or DBP) measurement in the time-dependent covariate models. ^ζ^Elevated total plasma cholesterol (Hypercholesterolemia) defined as total plasma cholesterol > 5.2 mmol/L; measured at the time of first available SBP or DBP in time-constant models, and coincident with each SBP or DBP measurement in the time-dependent covariate models. ^§^Antimalarials include chloroquine and hydroxychloroquine; defined as used ever during the study in time-constant models, and coincident with each TC (or SBP or DBP) measurement in the time-dependent covariate models. ^¥^Immunosuppressives include methotrexate, azathioprine, mycophenolate mofetil, cyclosporine and cyclophosphamide; defined as used ever during the study in time-constant models, and coincident with each TC (or SBP or DBP) measurement in the time-dependent covariate models.

In time-dependent models (Table 5, columns 3 to 5 inclusive), *mean *(HR = 1.025; *P *= 0.004) and *AM *(HR = 1.024; *P *= 0.004) SBP at each visit were significantly associated with a CAD event, with the same HR for each of these summary measures of SBP. In these models, other significant covariates included male sex (HR = 1.87; *P *= 0.04, for recent SBP model), age (HR = 1.06; *P *< 0.0001 for most *recent *SBP, HR = 1.04; *P *< 0.0001 for *mean *and *AM *SBP), SLEDAI-2K score (HR = 1.09; *P *< 0.0001 for all three models), and elevated cholesterol (HR = 1.72 for most *recent*, 1.68 for *mean*, and 1.69 for *AM *SBP; *P *= 0.03 for all three models) at each visit. *AUC *SBP (Table 5, last column) was not significantly associated with CAD.

### DBP models

Results of the proportional hazards models for CAD outcomes using various measures of diastolic blood pressure (DBP) were similar to the SBP models and are presented in Table 6.

**Table 6:** Proportional hazards models for coronary events using various measures of diastolic blood pressure

	Time-constant models				Time-dependent models							
	First available ('remote') DBP		Average of first two DBP		Most recent DBP		Mean DBP		Time-adjusted mean DBP		AUC DBP	
	HR (95% CI)	*p*	HR (95% CI)	*p*	HR (95% CI)	*p*	HR (95% CI)	*p*	HR (95% CI)	*p*	HR (95% CI)	*p*
Diastolic blood pressure (DBP)	1.00 (0.98, 1.02)	0.87	1.01 (0.99, 1.03)	0.46	1.02 (1.003, 1.05)	0.02	1.04 (1.01, 1.07)	0.02	1.04 (1.01, 1.07)	0.02	1.00 (1.00, 1.00)	0.94
Male sex	2.01 (1.13, 3.60)	0.02	2.00 (1.12, 3.58)	0.02	1.85 (1.02, 3.36)	0.04	1.72 (0.94, 3.15)	0.08	1.70 (0.93, 3.11)	0.09	1.88 (1.03, 3.43)	0.039
Age^ψ^	1.05 (1.04, 1.07)	< 0.0001	1.05 (1.04, 1.07)	< 0.0001	1.06 (1.04, 1.08)	< 0.0001	1.06 (1.04, 1.08)	< 0.0001	1.06 (1.04, 1.07)	< 0.0001	1.06 (1.04, 1.08)	< 0.0001
SLEDAI-2K^¶^	1.03 (1.01, 1.06)	0.01	1.03 (1.01, 1.06)	0.01	1.09 (1.05, 1.13)	< 0.0001	1.09 (1.05, 1.13)	< 0.0001	1.09 (1.05, 1.13)	< 0.0001	1.09 (1.05, 1.13)	< 0.0001
Elevated cholesterol^ζ^	1.42 (0.67, 3.00)	0.36	1.40 (0.66, 2.96)	0.38	1.70 (1.05, 2.74)	0.03	1.70 (1.06, 2.75)	0.03	1.69 (1.04, 2.73)	0.03	1.86 (1.16, 2.99)	0.01
Corticosteroids^β^	2.81 (1.16, 6.80)	0.02	2.74 (1.13, 6.63)	0.03	1.75 (0.99, 3.10)	0.054	1.76 (1.00, 3.11)	0.051	1.72 (0.97, 3.03)	0.06	1.81 (1.02, 3.19)	0.04
Antimalarials^§^	0.57 (0.36, 0.90)	0.02	0.59 (0.37, 0.94)	0.03	0.95 (0.60, 1.51)	0.82	0.96 (0.60, 1.53)	0.85	0.96 (0.60, 1.53)	0.85	0.92 (0.58, 1.47)	0.73
Immunosuppressives^¥^	0.91 (0.58, 1.43)	0.68	0.89 (0.56, 1.41)	0.61	1.45 (0.89, 2.34)	0.13	1.37 (0.85, 2.23)	0.20	1.38 (0.85, 2.23)	0.20	1.43 (0.88, 2.32)	0.14

95% CI: 95% confidence interval; AUC: area under the curve; BP: blood pressure (mmHg); DBP: diastolic blood pressure (mmHg); HR: hazard ratio; NS: not significant; *p: *p value; SBP: systolic blood pressure (mmHg); TC: total plasma cholesterol (mmol/L).

^ψ^Years; age at the time of first available TC (or SBP or DBP) in time-constant models and coincident with each TC (or SBP or DBP) measurement in the time-dependent covariate models. ^¶^SLE Disease Activity Index 2000; scores range from 0 to 105, with higher scores indicating more active disease; measured at the time of first available TC (or SBP or DBP) in time-constant models, and coincident with each TC (or SBP or DBP) measurement in the time-dependent covariate models. ^¢^Hypertension defined as Diastolic BP ≥ 90 or Systolic BP ≥ 140 mmHg; defined as present ever during the study in time-constant models, and coincident with each TC measurement in the time-dependent covariate models. ^β^Corticosteroids defined as used ever during the study in time-constant models, and coincident with each TC (or SBP or DBP) measurement in the time-dependent covariate models. ^ζ^Elevated total plasma cholesterol (Hypercholesterolemia) defined as total plasma cholesterol > 5.2 mmol/L; measured at the time of first available SBP or DBP in time-constant models, and coincident with each SBP or DBP measurement in the time-dependent covariate models. ^§^Antimalarials include chloroquine and hydroxychloroquine; defined as used ever during the study in time-constant models, and coincident with each TC (or SBP or DBP) measurement in the time-dependent covariate models. ^¥^Immunosuppressives include methotrexate, azathioprine, mycophenolate mofetil, cyclosporine and cyclophosphamide; defined as used ever during the study in time-constant models, and coincident with each TC (or SBP or DBP) measurement in the time-dependent covariate models.

In each of the multiple regression analyses presented in Tables 3 through 6, antiphospholipid antibodies, diabetes, and smoking were consistently statistically insignificant and therefore removed from the final models to maximize statistical power. Data on antihypertensive use and lipid-lowering therapy were incomplete for a proportion of visits. In subgroup analysis of these smaller datasets, neither antihypertensives nor lipid-lowering medications were significantly associated with CAD events (data not shown).

## Discussion

Through the use of proportional hazards regression modeling in a large sample of more than 950 patients, in whom collectively more than 18, 000 serial measurements of TC and BP were taken over a mean duration of 6.3 years, we were able to demonstrate and quantify the association between several important risk factors and CAD events in SLE.

Foremost, this study highlights the important role of traditional risk factors such as elevated TC and BP in SLE-related CAD and demonstrates a continuum of risk associated with these variables across the range of possible values they may assume. Previous studies have shown that traditional risk factors, such as hypercholesterolemia and hypertension, account for only a small proportion of the increased risk of CAD in SLE. However, in these studies, TC and BP were measured at baseline, in keeping with the premise of the Framingham model. Here we have shown that this remote measure of exposure to TC, SBP, or DBP is not predictive of CAD outcome among patients with SLE. Furthermore, recent measurements of TC and BP, which are also taken at a single point in time, are not able to quantify CAD risk to the same degree as "summary measures, " which capture cumulative exposure to these risk factors over the course of disease. We have previously shown that, unlike the general population, wherein TC and BP "track" over time, in patients with SLE, these risk factors take a dynamic course, varying because of changes in disease activity and treatment [5].

In this study, cumulative exposure to TC, SBP, and DBP was measured by using three summary measures, arithmetic mean, time-adjusted mean (AM), and area-under-the-curve (AUC). Time-dependent proportional hazards regression models were then applied to these summary measures. In this way, a sense of cumulative exposure was captured in two ways: first, in the form of a summary measure, and second, by determining the hazard related to this summary measure for an interval just before each and every sequential visit. In these time-dependent models, a sense of cumulative exposure to other covariates, including disease activity score, corticosteroids, and antimalarials, was also captured through the use of serial measurements of these variables, updated from one visit to the next.

For each of TC, SBP, and DBP, mean and AM summary measures were significantly predictive of a CAD event. In addition, the HR and accompanying *P *value of mean summary measures was the same as for AM summary measures in the case of each of TC, SBP, and DBP. This is likely related to the fact that overall, in this context, in which measurements were taken frequently, mean and AM values were very similar for each patient. However, when applied to a setting in which measurements are more irregular and infrequent, the AM, which is weighted for the interval between measurements, may be expected more accurately to reflect cumulative risk exposure and hence the overall risk of CAD. In the case of TC, a hazard ratio of 2.07 (*P *= 0.003) means that for every 1 mmol/L increase in mean (or AM) plasma TC level, the hazard of a CAD outcome increases 2.07-fold. In the case of SBP, a hazard ratio of 1.025 means that for every 1-mm Hg increase in mean SBP, the hazard of CAD outcome increases 1.025-fold.

Likewise, in the case of DBP, a hazard ratio of 1.04 means that for every 1-mm Hg increase in mean (or AM) DBP, the hazard of CAD outcome increases 1.04-fold. SBP and DBP are highly correlated, and, based on our analyses, either one or the other may be used to quantify CAD risk in patients with SLE.

AUC is very closely tied to length of follow-up, and for any given variable may only become larger over time. As such, it does not provide a sense of rise and fall in the variable of interest. Furthermore, it is not measured in the original units of the variable from which it is derived. These reasons may underlie the lack of association between AUC measures and CAD outcomes among SLE patients in our study.

In this proof-of-concept study, our chosen lipid marker of CAD risk was TC. In general, low-density lipoprotein cholesterol (LDL-C) is deemed the primary target of lipid-lowering therapy [11]. In the Toronto lupus cohort, measurement of nonfasting TC has been routine practice at every visit since 1975. However, measurement of lipid and lipoprotein subfractions is a more-recent addition to the data-collection protocol and performed only once yearly because of the need for a fasting sample. To derive summary measures and test their ability to quantify CAD risk, we required a very large dataset inclusive of a relatively large number of CAD outcomes. The TC and BP datasets fulfilled these methodologic requirements. In future, summary measures derived in this study may be applied to other risk factors, such as LDL-C.

How many measurements are enough to provide a valid summary measure, and how often should these measurements be taken in patients with SLE? In this study, the average of first two TC (or SBP or DBP) measurements was not significantly predictive of CAD outcome. Therefore, ideally three or more serial measurements should be sought. Although in this study, the mean gap between measurements used to calculate summary measures was 4.3 months, patients in whom the gap between one or more serial measurements exceeded 18 months were not included in the analyses. Further studies are required to determine the optimal number and frequency of measurements of TC and BP in evaluating CAD risk in SLE.

This study has provided several important insights into the role of demographic-, disease-, and treatment-related variables in SLE-related CAD. The most noteworthy are the increased risk of CAD with increasing disease activity and corticosteroid use, and the protective effect of antimalarials.

We found that for every unit increase in recent SLEDAI-2K disease activity score, the risk of CAD event by the time of the subsequent visit increased almost 10%. An increase in SLEDAI-2K score of 4 or more is generally deemed clinically significant [12]. This means that a minimum clinically significant increase in recent disease activity score is associated with approximately 46% increase in risk of CAD in the interval between sequential visits. Ibanez *et al*. [13] previously showed that for every unit increase in the time-adjusted mean *SLEDAI-2K *score (AMS), the hazard of a CAD outcome increases 1.08-fold. Collectively, these associations highlight the underpinning role of inflammation in SLE-related CAD.

Our patients with CAD events had greater disease activity at baseline and during follow-up, manifest in a multitude of organ systems, including musculoskeletal, cutaneous, renal, neural, vascular, and serosal. As the overall prevalence of chronic renal impairment was low and did not differ among groups with and without CAD, the association between disease activity and events seen in this study cannot be attributed to nephritis or renal impairment alone.

In studies of coronary risk factors in SLE, it is often difficult to tease apart the effect of corticosteroids from disease activity and traditional cardiac risk factors. In previous studies, longer duration of steroid use has been shown to be an independent risk factor for CAD in SLE [1,14]. With a retrospective chart review method, Karp *et al*. [15] showed that a 10-mg increase in the average daily prednisone-equivalent dose in the preceding year is independently associated with a 16% increase in the estimated 2-year CAD risk. Here, we quantified the CAD risk in patients with SLE by using prospectively collected data. Patients with CAD events received a significantly greater cumulative dose of corticosteroids during follow-up than did those who remained CAD free. In the regression models for first-available and average-of-first-two TC levels, exposure to corticosteroids at any time during the course of disease, irrespective of dose and duration of use, was associated with a dramatic 4.17-fold increased risk of a CAD event, independent of other risk factors. This HR was reduced to 1.85, but remained substantial and statistically significant, in the time-dependent models wherein recent exposure to corticosteroids was related to a CAD event.

In time-constant models for TC and BP, antimalarial use during the course of SLE was associated with a remarkable 50% to 59% reduction in hazard of a CAD event. The lack of a significant association between recent antimalarial use and CAD in the time-dependent models may point to a beneficial effect with long-term rather than short-term use. In previous studies, use of antimalarials has been associated with a reduction in TC level [5,14,16,17]. However, ours is one of only a few studies in which the use of antimalarials has been linked with a reduction in the risk of actual CAD events [18,19]. Furthermore, the halving of coronary risk makes a strong case for the use of antimalarials in patients with SLE, not only to control disease activity but also for cardioprotection.

As in previous studies, we have shown that male sex and older age are associated with increased hazard of a CAD event. In previous studies, anti-phospholipid antibodies, diabetes, and smoking were shown to be independent risk factors for CAD events in SLE [20]. However, in this study, in multiple regression analysis, such an association was not found, possibly because of the limited number of patients who smoke or have diabetes or anti-phospholipid antibodies. This study is not an exhaustive evaluation of traditional risk factors, and the role of other variables, such as family history of ischemic heart disease, body mass index (BMI), and waist/hip ratio, merit further investigation in future studies.

In summary, we showed that elevated TC and BP are both potentially treatable risk factors for CAD in SLE. Our study highlighted the importance of frequent measurements of BP and TC in management of patients with SLE. Although clinicians might consider the need for change in treatment based on single TC and BP measurements, their decision actually to do so should be made on the basis of the mean of several measurements.

## Conclusions

Overall, this study has both conceptual and practical significance. From a conceptual point of view, our findings illustrate that in a systemic inflammatory disease, for a dynamic risk factor such as TC or BP, summary measures such as mean and AM better reflect cumulative exposure and hence better quantify CAD risk. This is an important consideration in future studies of dynamic risk factors for CAD in a chronic relapsing-remitting disease such as SLE and may be used to derive risk-prediction models specifically for SLE. From a practical point of view, this study has shown that assessment of coronary risk related to TC and BP in patients with SLE relies on serial measurement of these risk factors throughout the relapsing-remitting course of SLE. Additionally, this study has shown that disease activity and corticosteroid use are CAD risk factors in SLE, indicating that disease activity should be recognized and optimally controlled by using the minimal effective dose of corticosteroids. The demonstration of a cardioprotective association of antimalarials highlights the staple role of this class of drugs in the management of patients with SLE. Finally, this study has quantified the risk associated with exposure to TC, SBP, and DBP over time, emphasizing the importance of these potentially treatable traditional risk factors in patients with SLE. Future efforts must be directed toward determining TC and BP cut-points for CAD risk stratification specifically among patients with SLE and the role of treatment of risk factors in reducing the incidence of CAD events.

## Abbreviations

ACR: American College of Rheumatology; AM: time-adjusted mean; AMS: adjusted mean SLE disease activity index 2000; AUC: area under the curve; BP: blood pressure; CAD: coronary artery disease; CI: confidence interval; DBP: diastolic blood pressure; ECG: electrocardiographic; HR: hazard ratio; LDL-C: low-density lipoprotein cholesterol; Max: maximum; Min: minimum; mm Hg: millimeters of mercury: mmol/L; millimoles per liter; MI: myocardial infarction; SBP: systolic blood pressure; SD: standard deviation; SLE: systemic lupus erythematosus; SLEDAI-2K: Systemic Lupus Erythematosus Disease Activity Index 2000; SLICC/ACR DI: Systemic Lupus Erythematosus International Collaborating Clinics/American College of Rheumatology Damage Index; TC: total cholesterol.

## Competing interests

The authors declare that they have no competing interests.

## Authors' contributions

MN participated in the study design, collection and analysis of data, interpretation of results, and preparation of the manuscript. DDG and MBU participated in the study design, collection of data, interpretation of results, and preparation of the manuscript. DI participated in the study design, analysis of data, interpretation of results, and preparation of the manuscript. PJH participated in the study design, interpretation of results, and preparation of the manuscript. All authors read and approved the manuscript for publication.
